# The impact of digital transformation on enterprise performance: An empirical analysis based on China’s manufacturing export enterprises

**DOI:** 10.1371/journal.pone.0299723

**Published:** 2024-03-06

**Authors:** Yunpei Wang, Tao Wang, Qingnian Wang

**Affiliations:** 1 School of Economics and Management, South China Normal University, Guangzhou, Guangdong, China; 2 School of Economics and Finance, South China University of Technology, Guangzhou, Guangdong, China; 3 School of International Education, South China University of Technology, Guangzhou, Guangdong, China; University of Central Punjab, PAKISTAN

## Abstract

Currently, countries worldwide are embracing digital strategies, enabling enterprises to utilize digital technology, digital supply chains, blockchain, and additional digital measures to increase their competitiveness. This paper analyzed the correlation between the digital transformation of manufacturing export enterprises and their business and export performance, focusing on China’s manufacturing export enterprises through empirical analysis. The study investigated the influence of digital transformation on enterprise performance. Using the Resource Based View theory and Trade theory, hypotheses were proposed and regression models were developed to analyze a sample of 1007 enterprises listed on the Shanghai and Shenzhen Stock markets from 2012 to 2019. The study conducted regression analysis, intermediate effect test, robustness test, stage lag, and heterogeneity analysis. The study found that (1) Manufacturing export enterprises listed in the stock market implemented digital transformation, leading to a significant positive impact on their overall performance. (2) Digital transformation led to cost reduction, improved R&D intensity, and enhanced human resources, among other benefits for enterprise performance. (3) According to the fractal analysis, non-state-owned enterprises exhibited more favorable effects on enterprise performance, and the digital transformation of manufacturing export companies in developed regions had a more significant impact on their performance. Finally, the study’s empirical results yielded pertinent proposals for digital transformation.

## Introduction

Recognizing the strategic importance of digitalization and transformation, China is working diligently to advance the design of top-level digital development. These efforts are driving industrial integration and exploring new drivers of economic growth. In general, Chinese global enterprises are pursuing two goals: improving production efficiency through the use of intelligent machines, and building digital platforms to improve operational efficiency and service quality systematically. By combining digital economy development strategies with those of other countries worldwide, domestic digital infrastructure investment and construction have been accelerated. Barriers to data flow between nations were gradually removed, resulting in a significant reduction in the cost of mutual investment in the digital economy. In addition, digital technology could be thoroughly integrated into the traditional economy, using third-party data for networks and products operated by third-party groups. Advanced technologies, such as big data and artificial intelligence, enable multi-dimensional correlation analysis to understand their customers and networks better than the operators’ customers. This correlation helps combine customer scenario challenges, solution value, and product competitiveness, leading to a more thorough analysis.

Digital transformation is of great importance for the manufacturing industry, especially for the developing countries that are increasingly dominating it. This transformation will lead to many new business models and products that will increase the power of manufacturing and exporting companies. In addition, greater value realization is expected [1]. As a major manufacturing nation, China has a robust industrial infrastructure to promote the digital economy actively. By implementing enterprise digitalization, it is possible to significantly reduce production costs, enhance enterprise research and development efforts, increase innovation potential, and promote high-quality growth in the manufacturing sector. Therefore, exploring the impact mechanism of digital transformation on the export performance and business performance of China’s manufacturing export enterprises, as well as strategies to promote the deep integration of digital transformation and the manufacturing industry and enhance the competitiveness of manufacturing export enterprises, is imperative in the digital era.

This study developed digital transformation metrics, using data from listed enterprises from 2012 to 2019, to examine the impact of digital transformation on enterprise performance. Specifically, it focused on the current status and challenges of manufacturing export enterprises in China in their digital transformation efforts and assessed the relationship between these enterprises’ business and export performance in terms of digital transformation.

The paper makes the following contributions: First, it comprehensively analyzes the current status of digital transformation and the operational and export performance of China’s manufacturing export enterprises. It also explores the problems manufacturing export enterprises face in digital transformation and manufacturing industry integration. Second, this paper establishes a theoretical model based on relevant theoretical analysis and enterprise-level data from the manufacturing industry. It also develops an appropriate empirical model to explore the impact of digital transformation on enterprises and investigates the relationship between the degree of digital transformation and enterprise operation and export performance. Finally, it provides recommendations to improve the performance of China’s manufacturing export enterprises in the context of digital transformation.

To study this, this paper covered the materials and methods, which included a literature review, theoretical analysis, theories and hyperthesis, and results and discussion, which included the empirical analysis and result analysis. Finally, this paper concluded with the academic and practical implications and the limitations and opportunities for the future.

## Materials and methods

### Literature review, theoretical analysis

#### Definition of concepts related to informatization, internalization, digitization, and digital Transformation

- **Informatization**

Generally, informatization refers to the construction of information systems in IT. According to Wikipedia, informatization involves the development and transformation of regions, economies, and societies through information technology and resources. The government’s report on informatization describes it as a historical process that utilized information technology and resources to enhance the quality of economic growth and facilitate social transformation. Some scholars have defined informatization as a business process carried out in the physical world that is supported by information systems. Informatization capability reflects a company’s effective management and utilization of information [2]. The rapid development of enterprise informatization construction has prompted Chinese companies to implement modern management systems, conduct more business operations online, and adopt standardized and scientific management and operation modes. This has led to a significant reduction in management and operation costs, and improved efficiency. To maximize the use of information technology resources, the previous information governance model focused on utilizing IT for effective company management [3].

- **Internetization**

There is no concrete and strict definition of "Internetization". As a result, there is often confusion between "Internet thinking" and "Internetization" [4]. The State Council has defined "Internet Plus" as the deep integration between the economy, society, and internet innovation. It aims to enhance the production and innovation capacity of the real economy and create an Internet-based development pattern. The transformation process of the real economy and traditional fields using Internet technology and primary business forms is at the core of Internetization [5]. When attempting to innovate on the Internet, enterprises must allocate the appropriate space and resources for trial and error. Having highly skilled technical personnel available is also critical to ensure effective planning and execution of business ventures.

- **Digitization**

Enterprises and individuals are transforming the physical world into the digital realm through various digital technologies, such as the Internet of Things, blockchain, big data, and mobile internet. This paradigm shift is not only advancing technology but also changing the ways people think. The characteristics of digitalization are as follows: (1) Digitalization and other technologies are used to build the physical world into the digital world. (2) Most activities and interactions conducted by human beings occur in the digital world, while a small amount of command and decision-making information returns to the physical world to control and operate devices and machines. (3) Digital data serves as the medium and carrier that connects the physical and digital worlds, providing the digital world’s foundation. During the digital transformation, it was necessary to change the enterprise’s organizational structure to promote technology-driven progress and facilitate the impetus of spontaneous innovation [6].

- **Digital transformation**

Digital transformation is rooted in digitalization, which is advancing in all areas. The study of digital transformation has become a progressively significant area of interest for academics and businesses alike. According to Google, digital transformation is the capacity to reimagine and redefine interactions with customers, partners, and employees using cutting-edge technology. The digital transformation of enterprises involves modernization, the development of new business models, and the introduction of new products and services for customers. According to the China Academy of Information and Communications Technology, digital transformation involves the comprehensive integration of industry and digital technology to improve efficiency. Digital technology is specifically used to achieve the digitalization of various elements and connections in the industry, leading to the optimization of resource allocation and business processes, while changing production methods to increase industrial efficiency.

#### Literature on the impact of digital transformation on enterprise performance

- **Literature on the digital transformation of the manufacturing industry**

Organizational change theory posits that digital transformation consists of measures taken by enterprises to adapt to external factors. Zaoui et al. contended that digital transformation altered customer relationships, internal processes, and value creation for enterprises, highlighting the need for successful leadership in this area [7]. He et al. investigated the effect of digital transformation on the underlying mechanisms and boundary conditions of green innovation. They found that digital change has a positive impact on substantive innovation, and they explored the boundary conditions of the impact of digital change on green innovation by analyzing the moderating effect of environmental orientation and separating the motivations into voluntary and mandatory [8]. Kumar et al. identified the barriers of supply chain digitizationin light of the Sustainable Development Goals (SDGs), and they found that the most crucial factor in the adoption of SCD was ‘administrative barriers.’ By establishing the relationship between SCD barriers and their impact on the SDGs, they sought to provide structural thinking and frameworks to assist supply chain managers in their decision-making process [9].

- **Literature on digital transformation measurement index**

Until now, the measurement standard of digital transformation index was not clear, and there was little research. In contrast, scholars’ research on digital transformation mainly focused on information technology capability, informatization level, and other aspects. Cooper et al. considered the economic benefits mainly measured by export profit, export income, export value, and so on [10]. Bharadwaj divided IT resources into IT infrastructure and IT human resources to measure the IT capability of the organization [11]. Peppard et al. summarized information technology capability into information infrastructure, management capability, and business alignment capability [12]. Aral et al. found that IT investment had no significant impact on ROA and net profit margin [13]. Nylen et al. believed that the enterprise’s good products were efficient, easy to learn, and the value of consumers’ need, so such digital innovative products can meet the trend of the retail consumer market [14].

- **Literature on enterprise performance measurement indicators**

Most of the studies on enterprise performance started from three perspectives: enterprise operating performance, enterprise innovation performance, and enterprise export performance. Some scholars took corporate social responsibility as business performance indicator [15]. Wang et al. used the return ratio on total assets and operating profit rate to measure corporate transformation performance [16]. Some authors choose to construct an index evaluation system to measure the business performance of enterprises. Kauffman et al. focus on the relationship between the digital economy and the innovation performance of enterprises. They believed that the application of digital technology could not only reduce the information friction between enterprises and the market, but also improve scientific decision-making [17]. Correa believed that the higher the level of market integration, the higher the level of innovation performance of enterprises [18]. Kanuri et al. empirically investigated the impact of an online sales push’s impact on salespeople’s effort allocation and sales performance, and their results indicated that following an online sales push, salespeople expended their effort based on a customer’s online propensity and potential before the push [19].

- **Literature on the path of digital transformation**

Berman et al. believed that the digital transformation of enterprises was the reconstruction of business models to improve the market competitiveness of enterprises [20]. Lerch believed that digital transformation could enhance the quality of products and services based on improving operational efficiency and ultimately increase the market share and influence of the enterprise [21]. Zhang et al. conducted an empirical analysis on 254 enterprises in Guangdong Province and found that both dimensions of extensive data capability have a significant positive impact on enterprise performance. The integration and utilization of extensive data resources can improve enterprise performance by positively affecting organizational learning [22]. Lai et al. show that digital transformation can reduce enterprise costs and improve enterprise service efficiency [23]. Qi et al. analyzed the influence of digitalization level on enterprise performance by establishing digital indicators based on the data of Chinese manufacturing enterprises from 2011 to 2018. They concluded that sales and management activities had two influence paths, and the results were insignificant because they offset each other [24]. Lee et al. addressed six dominant topics: smart factories, sustainability, and product-service systems, construction digital transformation, public infrastructure-centric digital transformation, techno-centric digital transformation, and business model-centric digital transformation. Their study contributed to adopting and demonstrating the ML-based topic modeling for intelligent and systematic bibliometric analysis [25]. Battisti et al. investigated the effects of technological and organizational change (T&O) on jobs and workers. They showed that firms that adopted T&O offered routine workers retraining opportunities to upgrade to more abstract jobs [26]. Jauhar et al. examined the application of digital transformation technologies in the related industry and analyzed product returns in the e-commerce industry [27].

- **Literature on the effect of digital transformation on enterprise performance of manufacturing export enterprises**

By adopting appropriate organizational change strategies, enterprises could change the aspects of administrative personnel, organizational tasks, and organizational technology. The specific methods and techniques have been improved and planned [28–31]. Walter et al. believed that enterprises also knew that they could successfully adjust their organizational structure only through organizational learning, dynamic changeability, and information technology [32]. Chen et al. constructed an evaluation system from three aspects of technological change, organizational change, and management change to measure the ability of manufacturing enterprises to make digital transformation [33]. Alexandre et al. investigated the relevance of some performance indicators of airline management and operational efficiency. By analyzing these performance indicators, it was possible to identify strategies to support decision-making to improve airline operational efficiency [34]. Parsheera highlighted some state-level differences in digital access, skills, and infrastructure across India—as a basis for dispelling assumptions about the homogeneity and universality of India’s digital transformation. They drew attention to the varying levels of digital readiness within India and the need to account for these variations in the design and implementation of the country’s digital initiatives [35].

### Analysis of the status of the digitization of manufacturing export enterprises

#### Current situation of digital transformation of domestic and foreign enterprises

Most domestic enterprises have started to implement digital transformation. Among different industries, the digital level gap was significant.

- In terms of the overall domestic digital transformation in the country, nearly 70% of enterprises have started to implement digital transformation, and the rest are in the planning stage. In December 2018, Zhiding.com surveyed 500 enterprises or institutions from the government, manufacturing, finance, education, retail, and other industries, and found that about 14% of enterprises had completed the digital transformation and were in the optimization and innovation stage. About 14% of enterprises were in the process of digital transformation. About 41% of enterprises were conducting one-off trials or partial rollouts of digital transformation; and another 31% were in the wait-and-see or planning mode.
- In terms of industries, the digitalization level of ICT, communication media, finance, and insurance industries was relatively high. In contrast, the digitalization level of the real estate, construction, and agriculture industries was relatively low. According to the report on China’s digital economy released by McKinsey Global Institute in December 2017, the digitalization level of 21 industries in China was evaluated from five groups. The higher the story, the higher the digitalization level, and the TMT industry was at the top.

From the management level, the high level of digitalization is in technology application and model innovation, and the low level of digitalization is in user experience and product service. In October 2018, IDC surveyed enterprises in six key industries, including retail, education, and manufacturing, and evaluated the level of digitalization from seven management levels. The results show that the digitalization level of technology application and mode innovation is high, while the digitalization level of user experience and product service is low.

#### Domestic manufacturing export enterprises’ current digital transformation situation

According to the study of China Internet Development Report 2022, the industrial scale of China’s digital economy reached 50.2 trillion yuan in 2022, accounting for 41.5% of GDP, which was an essential driving force to stabilize the sustainable growth of China’s economy.

In recent years, in response to the requirements of the State Council and the State-owned Assets Supervision and Administration Commission on the digital transformation of China’s manufacturing enterprises in the 14th Five-Year Plan, manufacturing export enterprises have continued to improve their digital transformation capability. The digital transformation degree of export enterprises in each subsector of the manufacturing industry was calculated according to the classification of subsectors of manufacturing enterprises in Wind. The data are shown in Table 1, with six subsectors: Textile, garment, and apparel industry; Printing and recording media reproduction industry; Computer, communication, and other electronic equipment manufacturing industry; Cultural and educational, industrial and art, sports and entertainment equipment manufacturing industry; Petroleum processing, coking, and nuclear fuel processing industry; Railway, ship, aerospace, and other transportation equipment manufacturing industry with an overall digital transformation degree higher than 0.1.

**Table 1:** Distribution of digital transformation degree of each industry.

Industry	Digital transformation degree	Industry	Digital transformation degree
Agricultural and sideline food processing	0.074	Non-metallic mineral products	0.0411
Food manufacturing	0.0824	Ferrous metal smelting processing	0.0498
Textile	0.0852	Cultural, educational, industrial, sports, and entertainment products manufacturing	0.2323
Wine, refined tea manufacturing	0.0489	Nonferrous metal smelting and processing	0.0383
Textile and garment	0.1823	Petroleum processing, coking, and nuclear fuel processing	0.1056
Leather and its products	0.0664	Manufacturing of chemical raw materials and products	0.0462
Wood processing and rattan palm products	0.846	Electrical and equipment manufacturing	0.0802
Furniture manufacturing	0.0439	Computer, communication equipment manufacturing	0.1307
Paper and paper products	0.0478	Automobile manufacturing	0.0948
Printing and reproduction	0.1863	Pharmaceutical manufacturing	0.0491
Metal products	0.0608	Railway, ship, aviation equipment manufacturing	0.1058
Chemical fiber manufacturing	0.0755	Rubber and plastic products	0.059
General equipment manufacturing	0.0786	Special equipment manufacturing	0.0676
Instrumentation manufacturing	0.0779	Waste resources comprehensive utilization	0.0018

### Theories, hypothesis, and theoretical models

#### Basis of theories

- **Resource-based view theory**

In 1984, Wernerfelt formally proposed the resource-based view theory, marking the birth of enterprise core competitiveness. In order to analyze the distribution and utilization of resources in an enterprise and draw feasible strategic choices based on the analysis, Wernerfelt analyzed the relationship between profits and resources on the premise of proposing the resource-based economic analysis tool, and then examined how specific resources and enterprise resource status management strategies were viewed and analyzed over time [36]. Wernerfelt mainly studied four kinds of attractive resources, including machine capacity, customer loyalty, production experience, and technological leadership, and used the resource-based view to divide resources into two categories: supplementing existing resources and acquiring complementary resources with existing resources. Tang believed that digital transformation was driven by technological change within organizations and can become the source of competitive advantage for enterprises through emerging technologies [37].

- **Trade theory**

Traditional trade theory was based on the concepts of absolute advantage and comparative advantage. Adam Smith proposed the theory of decisive advantage in 1776, while David Ricardo proposed the theory of comparative advantage, which argued that the differences in production technology between countries determine relative costs. Therefore, two countries need not have absolute advantages in trading two types of products. According to the current research of other scholars, there were mainly two views on the impact of digital transformation on enterprise costs. Most scholars believe that digital transformation can significantly reduce enterprises’ production and operation costs to enhance competitiveness.

#### Research hypotheses

Based on resource-based view theory, trade theory, and previous studies by other scholars, this paper discusses the purpose and necessity of digital transformation of manufacturing enterprises, possible measures that enterprises can take to cope with future business risks, how these measures can improve enterprise performance and what benefits digital transformation will bring. According to the resource-based theory, human resources and R&D intensity should be evaluated. Therefore, this paper proposed hypotheses H1a and H1b.

Hypothesis H1a: There was a positive relationship between the level of enterprise digital transformation and enterprise business performance;
Hypothesis H1b: There was a positive relationship between enterprise digital transformation level and enterprise export performance.

Specifically, the core explanatory variable enterprise digital transformation level had a significant positive impact on enterprise performance, which meant that improving the digital transformation level would positively affect enterprise operation and export performance. Enterprise digital transformation may positively affect enterprise performance, and digital transformation may affect enterprise performance through other pathways. Therefore, this paper proposed hypotheses H2a to H4c to be tested.

Hypothesis H2a: There was a negative relationship between enterprise digital transformation degree and enterprise operating cost;
Hypothesis H2b: There was a positive relationship between enterprise digital transformation level and enterprise R&D intensity;
Hypothesis H2c: There was a positive relationship between enterprise digital transformation and enterprise human resources;
Hypothesis H3a: The lower the enterprise operating cost, the higher the enterprise operating performance and export performance;
Hypothesis H3b: The more robust the R&D intensity, the higher the business performance and export performance;
Hypothesis H3c: The more human resources an enterprise had, the greater its operating performance and export performance;
Hypothesis H4a: Operating costs play an intermediary role between enterprise digital transformation and the performance of enterprise operation and export;
Hypothesis H4b: R&D intensity plays a mediating role between enterprise digital transformation and the performance of enterprise operation and export;
Hypothesis H4c: Human resources plays an intermediary role between enterprise digital transformation and the implementation of enterprise operation and export.

When an enterprise implemented digital transformation, its organizational tasks, technologies, human resources, and other aspects changed, which ultimately affected its overall profitability.

Hypothesis H5: There was a time-lag effect between enterprise digital transformation and enterprise business performance and export performance

Because the early stage of enterprise digital transformation was relatively large, the impact of technological progress had not been fully revealed, making the effect of digital transformation on performance seem small. In addition, due to the relatively high short-term cost pressure, enterprise profitability will be reduced, and other operating project expenses may be crowded out. Therefore, digital transformation may have a lagged effect on business performance and export performance.

Hypothesis H6: The influence of enterprise digital transformation level on enterprise performance was heterogeneous at the regional and economic development level.

In the era of digital economy and significant data, computing power has been an essential foundation for the rapid development of the national economy and micro enterprises. With the acceleration of digital transformation and upgrading, the total amount of data in society has experienced explosive growth. At the same time, the demand for data storage, transmission, calculation, analysis, and application has increased significantly. Only by accelerating the construction of computing power can we effectively stimulate the innovation vitality of data elements and promote the process of digital industrialization, industrial digitization, and high-quality economic development.

#### The construction of enterprise performance evaluation index

When selecting indicators to measure the business performance of enterprises, scholars had different strategies that directly selected accounting indicators to measure the business performance of enterprises with explained variables [38–41]. Based on the objective analysis, collection, sorting, and judgment of the operating conditions of various industries in China, and mathematical statistics to calculate and formulate, this paper used principal component analysis to construct the business performance evaluation system of listed companies in China. The nine financial indicators are defined in Table 2. All the data in this paper were obtained from the Wind database and Guotai ’an database.

**Table 2:** Selected financial indicators.

Dimension	Indicators	Indicators Definition	data sources
Profitability	f1: Net earnings per share	Total share capital at the end of the year	Wind database and Guotai ‘an database
	f2: Return on equity	Net profit/net assets	
	f3: Total Assets growth rate	Total assets growth this year/total assets at the beginning of the year	
	f4: Main business profit margin	Main business profit/primary business income	
Solvency	f5: Current ratio	Current assets/current liabilities	
	f6: Asset-liability ratio	Total liabilities/total assets	
Development capacity	f7: Total assets turnover	Sales revenue/average total assets	
Growth ability	f8: Net profit growth rate	Net profit increase/last year’s net profit	
	f9: Growth rate of primary business income	Value added of primary business/Last year’s main business income	

#### Construction of theoretical model

From the above analysis of both the hypothesis and the enterprise’s performance index, this paper put forward the following theoretical models to analyze the relationship between the degree of digital transformation and the enterprise’s performance. Meanwhile, in order to clarify the enterprise’s performance, this paper divided the enterprise’s performance into two areas: the enterprise’s business performance and the enterprise’s export performance. Therefore, the theoretical models were divided into two spheres.

**Fig 1:**
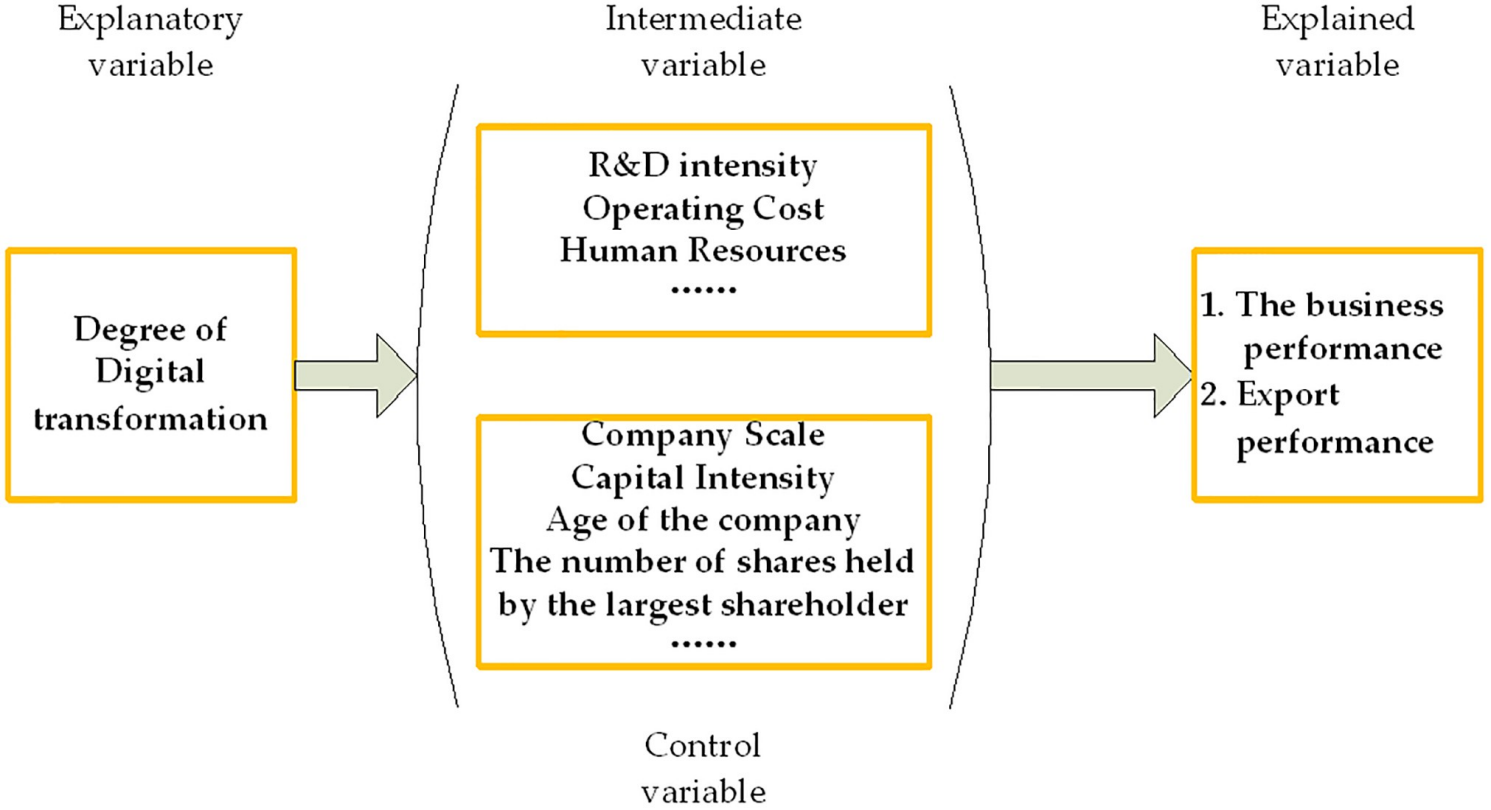
Illustration of the theoretical mode.

Therefore, the explained variables can be the business and the export performance of enterprises as measured by mathematical statistics and official standards. The core explanatory variables would mainly focus on the investment in digital technology, etc. The intermediary variables would be the ratio of R&D expenses to operating income to measure the R&D intensity and the percentage of operating costs to period expenses and re-expenses to calculate the company’s operating costs. The control variables would be the factors that can affect the business performance of enterprises, such as enterprise size, capital intensity, age, and the proportion of the largest shareholder, and so on. The illustration of the theoretical mode is shown in Fig 1.

## Results and discussion

### Empirical analysis

#### Data acquisition

The research object of this paper was 1007 listed companies in the Shanghai and Shenzhen stock exchanges from 2012 to 2019. Panel data was selected as the sample research object to reflect the financial indicators of each listed company, such as profitability, solvency, operating capacity, and growth capacity. Data such as the number of shares held by major shareholders, the proportion of independent directors, and the types of ownership of each listed company were obtained from the Wind database. Data reflecting the proportion of digital economy related part in the intangible assets details of each listed company in the total intangible assets at the end of the year and the yearly change were collected through the companies’ annual reports, announcements, corporate websites, and other channels.

The specific data samples were adjusted according to the following criteria: (1) complete data of manufacturing enterprises had been available for analysis for eight consecutive years; (2) the samples with special status and missing data were excluded. After a series of screening, the complete data of 1007 listed companies were finally obtained.

#### Variable selection

- Explained variables

This paper selected enterprises’ business performance and export performance as the explained variables, and used mathematical statistics methods to measure the official business performance standards.

- Core explanatory variables

With the help of the Wind database and Guotai ‘an database, this paper can easily collect the annual reports of many listed companies and obtain the corresponding data by browsing the official website. Therefore, according to the definition of digitalization and digital transformation, digital transformation pays more attention to the digital world rather than the physical world and pays more attention to the investment in digital technology. The core explanatory variable of this paper is the degree of digital transformation. There are two main ways to measure the level of digital transformation in existing studies: the first is the quantitative description method, which measures the degree of digital transformation by obtaining the cost of digital transformation [42]; of course, the second is the text analysis method, which measures the index by counting the frequency of keywords related to digital transformation in the text [43].

This paper adopted the first method for reference. First of all, with the help of the Wind database and Guotai ‘an database, this paper can easily collect many listed companies’ annual reports and obtain the corresponding data by browsing the official website. Secondly, the word frequency cannot effectively reflect the actual digital investment level of the company, and can only judge whether the enterprise has carried out big data transformation or the importance of digital transformation from the word frequency. Therefore, according to the definition of digitalization and digital transformation, digital transformation pays more attention to the digital world rather than the physical world and pays more attention to the investment in digital technology. By referring to the research of scholars such as He et al., the ratio of the intangible assets related to digital transformation in the intangible assets at the end of the annual report of listed companies is used to measure the level of digital transformation of enterprises. That is, if the intangible asset item contained keywords related to digital transformation technologies such as “management software”, “information management system”, “management system”, “intelligent platform”, “software system”, etc., the detailed item was defined as “digital intangible assets”. Then, the proportion of intangible assets in the current year was calculated by adding them. It was the proxy variable of the level of enterprise digital transformation [44].

- Intermediary variables

This paper used the ratio of R&D expenses to operating income to measure the R&D intensity of the company. It used the percentage of operating costs to period expenses and re-expenses to estimate the company’s operating costs which was easy to calculate and authoritative.

- Control variables

As for the control variables, this paper mainly selected the factors that can affect the business performance of enterprises as the control variables from the company level. Company size (Size), capital intensity (Capital), age (Age), and tpshahor (proportion of the largest shareholder) were selected as the control variables. In general, the larger the size and age of the company was, the stronger the ability to cope with external risks and other capabilities. Therefore, the above variables were selected as control variables, and the index selection was summarized in Table 3.

**Table 3:** Description of digital transformation regression model variables.

Variable type	Variable name	Variable symbol	Variable definition
Explained variable	The business performance	Prcomp	Calculated by using principal component analysis
	Export performance	Export	Export income from overseas business/income from the central business
Explanatory variable	Degree of Digital transformation	Transfer	The ratio of the digital transformation-related portion of the intangible asset detail item at the end of the annual report to the total intangible assets
Intermediate variable	R&D intensity	Inner	expenses/operating income
	Operating Cost	Cost	(operating cost + period expenses)/operating revenue
	Human Resources	Peor	Number of graduates/Total number of employees
Control variable	Company Scale	Size	Log of the total assets of the listed company at the end of the year
	Capital Intensity	Capital	fixed assets/Total employees
	Age of the company	Age	the year minus when the company went public
	The number of shares held by the largest shareholder	Tpshahor	Calculated this indicator based on the proportion of the largest shareholder

### Model construction

To further clarify the causal relationship of various variables and test the hypotheses, this paper set up a regression model to test the ideas, and according to the research hypothesis of enterprise digital transformation degree and enterprise performance, regression model 1 and regression model 2 were set up based on four control variables:

Model 1:

$$Prcomp_{i,t}=\alpha +\beta_{1}Size_{i,t}+\beta_{2}{Capital}_{i,t}+\beta_{3}Age_{i,t}+\beta_{4}Tpshahor_{i,t}+\varepsilon_{i,t}$$ (1)

Model 2:

$${Export}_{i,t}=\alpha +\beta_{1}Size_{i,t}+\beta_{2}{Capital}_{i,t}+\beta_{3}Age_{i,t}+\beta_{4}Tpshahor_{i,t}+\varepsilon_{i,t}$$ (2)

In the control variable group, digital transformation (Transr) was added, and model 3 and model 4 were established to verify hypotheses H1a and H1b, respectively.

Model 3:

$$Prcomp_{i,t}=\alpha +\beta_{1}Size_{i,t}+\beta_{2}{Capital}_{i,t}+\beta_{3}Age_{i,t}+\beta_{4}Tpshahor_{i,t}+\beta_{5}T\text{rans}{\text{r}}_{i,t}+\varepsilon_{i,t}$$ (3)

Model 4:

$${Export}_{i,t}=\alpha +\beta_{1}Size_{i,t}+\beta_{2}{Capital}_{i,t}+\beta_{3}Age_{i,t}+\beta_{4}Tpshahor_{i,t}+\beta_{5}T\text{rans}{\text{r}}_{i,t}+\varepsilon_{i,t}$$ (4)

To test hypotheses H3a, H3b, and H3c, based on model 1 and model 2, the substitution variables of Cost, R&D intensity, and human resources (Cost, Innor, and Peor) were added, and regression models 5 to 10 were established:

Model 5:

$$Prcomp_{i,t}=\alpha +\beta_{1}Size_{i,t}+\beta_{2}{Capital}_{i,t}+\beta_{3}Age_{i,t}+\beta_{4}Tpshahor_{i,t}+\beta_{5}Cost_{i,t}+\varepsilon_{i,t}$$ (5)

Model 6:

$$Prcomp_{i,t}=\alpha +\beta_{1}Size_{i,t}+\beta_{2}{Capital}_{i,t}+\beta_{3}Age_{i,t}+\beta_{4}Tpshahor_{i,t}+\beta_{5}Innor_{i,t}+\varepsilon_{i,t}$$ (6)

Model 7:

$$Prcomp_{i,t}=\alpha +\beta_{1}Size_{i,t}+\beta_{2}{Capital}_{i,t}+\beta_{3}Age_{i,t}+\beta_{4}Tpshahor_{i,t}+\beta_{5}Peo{\text{r}}_{i,t}+\varepsilon_{i,t}$$ (7)

Model 8:

$${Export}_{i,t}=\alpha +\beta_{1}Size_{i,t}+\beta_{2}{Capital}_{i,t}+\beta_{3}Age_{i,t}+\beta_{4}Tpshahor_{i,t}+\beta_{5}\text{Cos}{\text{t}}_{i,t}+\varepsilon_{i,t}$$ (8)

Model 9:

$${Export}_{i,t}=\alpha +\beta_{1}Size_{i,t}+\beta_{2}{Capital}_{i,t}+\beta_{3}Age_{i,t}+\beta_{4}Tpshahor_{i,t}+\beta_{5}Innor_{i,t}+\varepsilon_{i,t}$$ (9)

Model 10:

$${Export}_{i,t}=\alpha +\beta_{1}Size_{i,t}+\beta_{2}{Capital}_{i,t}+\beta_{3}Age_{i,t}+\beta_{4}Tpshahor_{i,t}+\beta_{5}Peo{\text{r}}_{i,t}+\varepsilon_{i,t}$$ (10)

To test the path analysis in hypothesis 2, this paper established the relationship between digital transformation (Transr) and the variables of cost, R&D intensity, and human resource substitution variables (Cost, Innor, and Peor). It established the basic regression model 11 to 13 to control the variables:

Model 11:

$$C\text{os}{\text{t}}_{i,t}=\alpha +\beta_{1}Size_{i,t}+\beta_{2}{Capital}_{i,t}+\beta_{3}Age_{i,t}+\beta_{4}Tpshahor_{i,t}+\varepsilon_{i,t}$$ (11)

Model 12:

$$I\text{nno}{\text{r}}_{i,t}=\alpha +\beta_{1}Size_{i,t}+\beta_{2}{Capital}_{i,t}+\beta_{3}Age_{i,t}+\beta_{4}Tpshahor_{i,t}+\varepsilon_{i,t}$$ (12)

Model 13:

$$Peo{\text{r}}_{i,t}=\alpha +\beta_{1}Size_{i,t}+\beta_{2}{Capital}_{i,t}+\beta_{3}Age_{i,t}+\beta_{4}Tpshahor_{i,t}+\varepsilon_{i,t}$$ (13)

Digital Transformation (Transr) was added to Model 11, and Model 14 was established to verify hypothesis H2a:

Model 14:

$$C\text{os}{\text{t}}_{i,t}=\alpha +\beta_{1}Size_{i,t}+\beta_{2}{Capital}_{i,t}+\beta_{3}Age_{i,t}+\beta_{4}Tpshahor_{i,t}+\beta_{5}T\text{rans}{\text{r}}_{i,t}+\varepsilon_{i,t}$$ (14)

Digital Transformation (Transr) was added to Model 12, and Model 15 was established to verify hypothesis H2b:

Model 15:

$$I\text{nno}{\text{r}}_{i,t}=\alpha +\beta_{1}Size_{i,t}+\beta_{2}{Capital}_{i,t}+\beta_{3}Age_{i,t}+\beta_{4}Tpshahor_{i,t}+\beta_{5}T\text{rans}{\text{r}}_{i,t}+\varepsilon_{i,t}$$ (15)

Digital Transformation (Transr) was added to Model 13, and Model 16 was established to verify hypothesis H2c:

Model 16:

$$Peo{\text{r}}_{i,t}=\alpha +\beta_{1}Size_{i,t}+\beta_{2}{Capital}_{i,t}+\beta_{3}Age_{i,t}+\beta_{4}Tpshahor_{i,t}+\beta_{5}T\text{rans}{\text{r}}_{i,t}+\varepsilon_{i,t}$$ (16)

Finally, based on models 3 and 4, intermediate variables, namely Cost, R&D intensity, and human resource substitution variables (Cost, Innor, and Peor), were added to complete the final model construction of path analysis. Models 17 to 22 were established to verify hypotheses H4a, H4b, and H4c:

Model 17:

$$Prcomp_{i,t}=\alpha +\beta_{1}Size_{i,t}+\beta_{2}{Capital}_{i,t}+\beta_{3}Age_{i,t}+\beta_{4}Tpshahor_{i,t}+\beta_{5}T\text{rans}{\text{r}}_{i,t}+\beta_{6}{C\text{os}{\text{t}}_{i,t}+\varepsilon }_{i,t}$$ (17)

Model 18:

$$Prcomp_{i,t}=\alpha +\beta_{1}Size_{i,t}+\beta_{2}{Capital}_{i,t}+\beta_{3}Age_{i,t}+\beta_{4}Tpshahor_{i,t}+\beta_{5}T\text{rans}{\text{r}}_{i,t}+\beta_{6}{I\text{nno}{\text{r}}_{i,t}+\varepsilon }_{i,t}$$ (18)

Model 19:

$$Prcomp_{i,t}=\alpha +\beta_{1}Size_{i,t}+\beta_{2}{Capital}_{i,t}+\beta_{3}Age_{i,t}+\beta_{4}Tpshahor_{i,t}+\beta_{5}T\text{rans}{\text{r}}_{i,t}+\beta_{6}{Peo{\text{r}}_{i,t}+\varepsilon }_{i,t}$$ (19)

Model 20:

$${Export}_{i,t}=\alpha +\beta_{1}Size_{i,t}+\beta_{2}{Capital}_{i,t}+\beta_{3}Age_{i,t}+\beta_{4}Tpshahor_{i,t}+\beta_{5}T\text{rans}{\text{r}}_{i,t}+\beta_{6}{C\text{os}{\text{t}}_{i,t}+\varepsilon }_{i,t}$$ (20)

Model 21:

$${Export}_{i,t}=\alpha +\beta_{1}Size_{i,t}+\beta_{2}{Capital}_{i,t}+\beta_{3}Age_{i,t}+\beta_{4}Tpshahor_{i,t}+\beta_{5}T\text{rans}{\text{r}}_{i,t}+\beta_{6}{I\text{nno}{\text{r}}_{i,t}+\varepsilon }_{i,t}$$ (21)

Model 22:

$${Export}_{i,t}=\alpha +\beta_{1}Size_{i,t}+\beta_{2}{Capital}_{i,t}+\beta_{3}Age_{i,t}+\beta_{4}Tpshahor_{i,t}+\beta_{5}T\text{rans}{\text{r}}_{i,t}+\beta_{6}{Peo{\text{r}}_{i,t}+\varepsilon }_{i,t}$$ (22)

In the above model, α represented the intercept, β represented the coefficient of each variable, and Ɛ defined the residual term.

#### Empirical analysis

- **Measurement of business performance**

In this paper, the collected indicator data were standardized by referring to the relevant studies for measuring enterprise business performance. Then, KMO and Barlett tests were conducted, and the KMO value after the tests was analyzed. The selected metric was well-suited for analysis if the value exceeded 0.8. It was more suitable for analysis if it was more significant than 0.7 and less than 0.8. It can be analyzed if it is more important than 0.6 and less than 0.7. If this value was less than 0.6, it was unsuitable for analysis. If the p-value corresponding to the Bartlett test was less than 0.05, it indicated that the principal component analysis was appropriate.

According to the enterprise operation performance evaluation system constructed in this paper, nine financial index data from 1007 listed companies were collected and sorted into matrix data sets. KMO and Barlett tests were conducted in Table 4.

**Table 4:** Performance KMO and Bartlett test results.

	-	Result value
KMO sampling suitability quantity	-	0.616
Bartlett sphericity test	p-value	0.000

The test results showed that the KMO value of the enterprise business performance index after the test was 0.616, higher than 0.6, p-value = 0.000&lt; 0.005, indicating that the data meet the requirements of principal component analysis.

The calculation results were as follows: As shown in Table 2, STATA16.0 was used to conduct principal component analysis on enterprise business performance, and the main components with an eigenvalue more significant than one were selected to obtain five main details, namely, Cop1, Cop2, Cop3, Cop4, and Cop5, which extracted a total of 83.230% of the index variables of 9 financial indicator variables. In addition, the variance percentage of each component reached more than 10%, and the principal component 1 accounted for 23.602%, the principal component 2 accounted for 16.647%, the principal component 3 accounted for 16.368%, the principal component 4 accounted for 15.390%, and the principal component 5 accounted for 11.223%, which had a good reflection of the whole sample. The principal component coefficients of each financial index in the enterprise performance evaluation system are shown in Table 5.

**Table 5:** Explanation and total variance of business performance indicators.

Component	Eigenvalue	Variance percent	Cumulative variance
f1	2.124	0.23602	0.23602
f2	1.498	0.16647	0.40249
f3	1.473	0.16368	0.56617
f4	1.385	0.15390	0.72007
f5	1.010	0.11223	0.83230
f6	0.949	-	-
f7	0.846	-	-
f8	0.783	-	-
f9	0.412	-	-

The principal component coefficients of business performance indicators are shown in Table 6.

**Table 6:** Principal component coefficients of business performance indicators.

variable	Cop1	Cop2	Cop3	Cop4	Cop5
Earnings per share	0.491	0.260	-0.157	0.084	0.078
Return on equity	0.188	0.197	-0.352	0.469	-0.155
Growth rate of total assets	0.036	0.069	0.716	0.360	-0.184
A profit margin of the leading business	-0.139	0.030	0.217	0.357	0.784
Current ratio	0.074	0.410	0.070	-0.192	0.343
Asset-liability ratio	-0.509	0.257	-0.011	0.102	-0.295
Turnover of total assets	-0.263	0.465	-0.258	0.099	0.031
Net profit growth rate	-0.017	0.186	0.143	-0.664	0.110
The growth rate of primary business revenue	0.191	0.326	0.374	-0.060	-0.307

The five principal component expressions can be obtained from Table 6 as follows:

$$\text{Co}{\text{p}}_{1}=0.491*f_{1}+0.188*f_{2}+0.036*f_{3}-0.139*f_{4}+0.074*f_{5}-0.509*f_{6}-0.263*f_{7}-0.017*f_{8}+0.191*f_{9}$$ (23)

$${\text{Cop}}_{2}=0.260*f_{1}+0.197*f_{2}+0.069*f_{3}+0.030*f_{4}+0.410*f_{5}+0.257*f_{6}+0.465*f_{7}+0.186*f_{8}+0.326*f_{9}$$ (24)

$$\text{Co}{\text{p}}_{3}=-0.157*f_{1}-0.352*f_{2}+0.716*f_{3}+0.217*f_{4}+0.070*f_{5}-0.011*f_{6}-0.258*f_{7}+0.143*f_{8}+0.374*f_{9}$$ (25)

$$\text{Co}{\text{p}}_{4}=0.084*f_{1}+0.469*f_{2}+0.360*f_{3}+0.357*f_{4}-0.192*f_{5}+0.102*f_{6}+0.099*f_{7}-0.664*f_{8}-0.060*f_{9}$$ (26)

$$\text{Co}{\text{p}}_{5}=0.078*f_{1}-0.155*f_{2}-0.184*f_{3}+0.784*f_{4}+0.343*f_{5}-0.295*f_{6}+0.031*f_{7}+0.110*f_{8}-0.307*f_{9}$$ (27)

Each index was multiplied by the variance contribution rate of the corresponding component, then divided by the cumulative total contribution rate of the five principal components, and finally summed to obtain the comprehensive score of enterprise business performance.

- **Descriptive statistics of study variables**

The descriptive statistical results of the indicators of digital transformation of manufacturing export enterprises, business performance, export performance, and other related variables are shown in Table 7.

**Table 7:** Descriptive statistical results of related variables such as business performance of manufacturing enterprises.

Name	Sample Size	Minimum	Maximum	Mean	standard deviation	median
Prcomp	8056	-12.772	1.626	0.056	0.226	0.062
Export	8056	-0.049	3.717	0.196	0.222	0.119
Transr	8056	0.000	1.746	0.079	0.133	0.035
Peor	8056	0.000	1.000	0.213	0.153	0.175
Innor	8055	-0.025	0.843	0.035	0.032	0.031
Cost	8056	0.191	15.689	0.928	0.221	0.933
Size	8056	0.690	1.000	0.810	0.045	0.804
Capital	8056	0.000	5.890	0.464	0.496	0.306
Age	8056	-3.000	27.000	10.330	6.546	9.000
Tpshahor	8056	0.030	0.891	0.332	0.143	0.311

- **Correlation analysis**

In order to preliminarily judge the correlation between the performance of manufacturing export enterprises, digital transformation, operating cost, research and development intensity, and other relevant variables and their degree, this paper conducted a correlation analysis of the above variables.

From the correlation test results in Table 8, it can be seen that there was a strong positive correlation between enterprise digital transformation and enterprise business performance indicators, and enterprise export performance indicators. H1a and H1b can be tentatively verified.

**Table 8:** Correlation coefficient matrix of research variables.

	1	2	3	4	5	6	7	8	9	10
prcomp(1)	1									
Export(2)	-0.006	1								
Transr(3)	0.035***	0.020***	1							
Peor(4)	0.037**	0.135**	0.144**	1						
Innor(5)	0.019**	0.050**	0.119**	0.367**	1					
Cost(6)	-0.217**	-0.004***	-0.009	-0.016	0.254**	1				
Size(7)	0.060**	-0.044**	-0.035**	0.116**	-0.142**	0.002	1			
Capital(8)	-0.034**	-0.028*	-0.129**	0.018	-0.093**	0.065**	0.344**	1		
Age(9)	-0.032**	-0.022*	0.016	0.063**	-0.154**	0.103**	0.446**	0.149**	1	
Tpshahor(10)	0.062**	-0.045**	0.024*	-0.014	-0.102**	-0.052**	0.142**	0.051**	-0.035**	1

*** means p < 0.01,

** means p < 0.05,

* means p < 0.1.

Before the following regression analysis, this paper conducted collinearity diagnosis for each regression model by calculating the variance inflation factor (VIF). If the value was less than 5, there was no collinearity problem. The results are shown in Table 9. The VIF values of all the models were less than 5, indicating that there was no collinearity problem among the variables:

**Table 9:** Collinearity diagnosis of variables.

Variable	VIF
Degree of Digital Transformation (Transr)	1.323
Operating Cost (Cost)	1.516
Research and Development Intensity (Innor)	1.883
Human Resources (Peor)	1.291
Company Size (Size)	1.443
Capital intensity (Capital)	1.552
Business Age (Age)	1.008
Largest shareholder (Tpshahor)	1.739

#### Regression analysis and hypothesis test

Through the correlation analysis in the above section, the relationship between digital transformation and various variables can be tentatively determined. To further explore the causal relationship of multiple variables, this paper used SPSS statistical analysis software to conduct regression analysis on 1007 company samples. It tested the assumptions in the previous section according to the model. The empirical results of digital transformation on the performance of manufacturing export enterprises are shown in Table 10.

**Table 10:** Results of empirical analysis of digital transformation of manufacturing export enterprises on enterprise performance.

Variables	Enterprise Performance (Prcomp)		Enterprise Export Performance (Export)	
	Model 1	Model 3	Model 2	Model 4
Constant	-0.362** (-7.111)	-0.368** (-7.222)	0.337** (6.677)	0.340** (6.748)
Size	0.531** (7.966)	0.533** (8.001)	-0.143* (-2.166)	-0.144* (-2.185)
Capital	-0.028** (-5.288)	-0.026** (-4.902)	-0.005 (-0.992)	-0.006 (-1.203)
Age	-0.002** (-5.441)	-0.002** (-5.546)	-0.001 (-0.753)	-0.001 (-0.691)
Tpshahor	0.075** (4.212)	0.073** (4.118)	-0.064** (-3.652)	-0.063** (-3.599)
Transfer	-	0.053** (2.800)	-	0.031*** (1.632)
R ^2^	0.014	0.018	0.012	0.021
Adjusted R ^2^	0.002	0.006	0.003	0.014
Number of Samples	8056	8056	8056	8056
F Value	23.646***	27.574***	6.502***	7.460***

*** means p < 0.01,

** means p < 0.05,

* means p < 0.1.

#### Lagged effect analysis

To test hypotheses H4a, H4b, and H4c, models 17 to 22 were constructed to test the mediation effect. According to the above empirical results, it was clear: (1) digital transformation (Transr) had a significant positive effect on business performance (Prcomp), digital transformation (Transr) had a significant positive effect on export performance, and digital transformation (Transr) hurt business cost (Cost). Digital transformation (Transr) had a significant positive effect on research and development intensity (Innor), and digital transformation (Transr) had a positive effect on human resources (Peor). In this part, Model 20, Model 21, and Model 22 were used to analyze whether the coefficient corresponding to digital transformation in the intermediary effect was significant.

It was analyzed above that enterprise digital transformation had a significant positive effect on enterprise performance (Prcomp and Export), research and development intensity (Innor), and human resources (Peor) and a significant negative effect on the company’s operating Cost (Cost). The mediating role of operating cost, R&D intensity, and human resources was examined. From Table 11, the R^2^ adjusted by model 17 was 0.015, which was 0.009 higher than that adjusted by model 3. The fit degree of the model was good and improved. The absolute value of the regression coefficient of the digital transformation of the independent variable on the business performance of the dependent variable decreased. However, it was still significant (β = 0.043, p<0.05), indicating that operating costs partially mediated the relationship between the level of digital transformation of enterprises and business performance.

**Table 11:** Results of mediating effect test and analysis of enterprise performance in the digital transformation degree of manufacturing enterprises.

Variables	Enterprise Performance (Prcomp)			Enterprise Export Performance (Export)		
	Model 17	Model 18	Model 19	Model 20	Model 21	Model 22
Constant	-0.116* (-2.258)	-0.357** (-6.953)	-0.350** (-5.715)	0.226** (3.749)	0.315** (6.197)	0.309** (6.181)
Size	0.457** (6.992)	0.528** (7.915)	0.496** (6.188)	-0.413** (-5.263)	-0.132* (-1.996)	-0.056 (-0.848)
Capital	-0.020** (-3.679)	-0.027** (-4.956)	-0.022** (-3.560)	0.006 (1.573)	-0.005 (-0.870)	-0.006 (-1.067)
Age	-0.001** (-3.549)	-0.002** (-5.711)	-0.003** (-5.149)	-0.007** (-11.918)	-0.005* (-0.314)	-0.002** (-0.714)
Tpshahor	0.060** (3.439)	0.070** (3.917)	0.087** (4.130)	-0.067** (-3.814)	-0.059** (-3.308)	-0.072** (-4.115)
Transr	0.043** (2.807)	0.050** (2.990)	0.047*** (2.137)	0.026*** (1.371)	0.023** (1.224)	0.028*** (3.472)
Cost	-0.213** (-19.003)	-	-	-0.046** (-2.698)	-	-
Inner	-	0.142** (1.767)	-	-	0.270** (3.395)	-
Peor	-	-	0.040** (2.014)	-	-	0.203** (12.432)
R ^2^	0.057	0.015	0.032	0.021	0.013	0.051
Adjusted R ^2^	0.015	0.008	0.012	0.017	0.005	0.023
Number of Samples	8056	8056	8056	8056	8056	8056
F Value	80.774***	20.222***	89.527***	18.909***	7.316***	31.283***

*** means p < 0.01,

** means p < 0.05,

* means p < 0.1.

Similarly, after adding the research and development intensity of the intermediate variables (Innor), digital transformation was significant; the absolute value of the regression coefficient at the 0.05 level decreased from 0.053 to 0.050, indicating that R&D intensity played a partial mediating role in the relationship between digital transformation and business performance. Digital transformation was significant after adding the intermediary variable of human resources (Peor). At the level of 0.05, the absolute value of the regression coefficient decreases to 0.047, indicating that human resources played a partial mediating role in the relationship between the level of digital transformation and business performance. Then, based on model 4, models 20 to 22 also met the above analysis and demonstration. Operating cost, R&D intensity, and human resources also partially mediated the relationship between digital transformation and the export performance of enterprises. In summary, digital transformation affected the business performance and export performance of enterprises through operating costs, R&D intensity, and human resources. Hypotheses H4a, H4b, and H4c were verified, and the results were valid.

### Result analysis

Based on the financial data of listed companies, this paper investigated the impact of digital transformation on the performance of manufacturing export enterprises. It analyzed and demonstrated the relationship between the two from theoretical and empirical aspects. This paper analyzed and summarized the selection and measurement of proxy variables of digital transformation degree and enterprise performance, which provided a theoretical basis for further analysis, and put forward six hypotheses and established relevant models, which were verified by empirical method (Table 12).

**Table 12:** Results of hypothesis empirical test.

Serial number	hypothesis descriptions	Test results
H1a	It is assumed that there is a positive relationship between digital transformation of enterprises and their business performance	supported
H1b	It is assumed that there is a positive relationship between the digital transformation of enterprises and their export performance	supported
H2a	It is assumed that there is a negative relationship between the digital transformation of enterprises and their operating costs	supported
H2b	It is assumed that there is a positive relationship between enterprise digital transformation degree and enterprise R&D intensity	supported
H2c	It is assumed that there is a positive relationship between enterprise digital transformation degree and enterprise human resources	supported
H3a	It is assumed that the lower the operating cost of the enterprise, the more support the business performance and export performance of the enterprise	supported
H3b	It is assumed that the stronger the R&D intensity of the enterprise, the more supportive the business performance and export performance of the enterprise	supported
H3c	It is assumed that the more human resources the enterprise had, the more support the business performance and export performance of the enterprise	supported
H4a	It is assumed that operating cost plays an intermediary role in supporting the relationship between the digital transformation of enterprises and their operating and export performance	supported
H4b	It is assumed that the R&D intensity plays an intermediary role in supporting the relationship between enterprise digital transformation and the performance of enterprise operation and export	supported
H4c	It is assumed that human resources play an intermediary role in supporting the relationship between enterprise digital transformation and the performance of enterprise operations and export	supported
H5	The hypothesis that there is a time-lag effect between the digital transformation of enterprises and their business performance and export performance is not supported.	Not supported
H6	It is assumed that heterogeneity and differences in regional and economic development levels support the influence of H6 enterprises’ digital transformation degree on enterprise performance	supported

This paper examined the impact of digital transformation of manufacturing export enterprises on the performance of enterprises. Through empirical research, it was found that: (1) In the analysis based on large samples, digital transformation of manufacturing export enterprises had a significant positive impact on the performance of enterprises. (2) The multiple regression analysis of the intermediary effect test found that digital transformation of enterprises can improve enterprise performance by improving enterprise human resources, increasing enterprise research and development intensity, and reducing operating costs. (3) By analyzing the nature of enterprise, it was found that the digital transformation of non-state-owned enterprises had a more positive impact on enterprises’ business performance and export performance, while non-state-owned enterprises had less. (4) By analyzing the region and economic development level, it was found that the digital transformation of enterprises in coastal areas had a significantly greater impact on enterprise performance more than in inland areas.

## Conclusions

### Academic implications

In the empirical aspect, the empirical research was conducted by using the relevant enterprise data collected and organized in the Wind database and Guotai’ an database. Multiple regression models were constructed to verify the hypotheses step by step, and the intermediary effect test method was used to determine the role of human resources, operating costs, and R&D intensity in the influence of the level of digital transformation on enterprise performance of manufacturing export enterprises. Then, the robustness of the model was verified by replacing the explained variables, and the possible lagged effect of digital transformation was explored. Finally, heterogeneity analysis was conducted from the dimensions of enterprise nature, region, and economic development.

This paper measured the digital transformation of enterprises by using the proportion of the total assets of digital-related parts in the intangible assets in the annual report to the total intangible assets, which had specific innovative significance for the study of digital transformation of enterprises. This paper also evaluated the impact of digital transformation on enterprise performance from the enterprise dimension, compared with the existing research, and provided valuable countermeasures and suggestions for enterprises in the digital transformation process. In addition, this paper used the data of more than one thousand listed manufacturing export enterprises in China to study the impact of digital transformation on the business performance of enterprises and selected a set of variable indicator data to analyze and study the manufacturing enterprises that occupied the first share of China’s GDP and were most affected by digital transformation, which had specific innovative significance.

### Practical implications

Based on the above study of digital transformation on enterprise performance, the following policy suggestions were made. First, enterprises should accelerate the pace of organizational change for digital transformation to actively seek to resume work and production, accurately control inventory, improve management and operational efficiency, and reduce enterprise operating costs. Second, enterprises should formulate digital technology research and implementation plans as soon as possible to open up the barriers between digital technology and the production environment, supply chain, market sales, etc. Third, the government should promote the development of digital transformation and strengthen the construction of relevant digital infrastructure to eliminate barriers to regional data and digital industries, promote the free flow of data elements, and introduce applicable data protection, collection, use, and transmission standards and guidance recommendations. Fourth, the government should emphasize the training and introduction of digital talents, cultivate technical and managerial skills with innovative thinking and skillful use and management of data information, and encourage enterprises to conduct specialized training for employees and introduce high-quality talents for management.

### Limitations

This study had several limitations. First, due to the limitations of data quantity, quality, and channel, this paper only analyzed the data of 1007 manufacturing export enterprises. Second, the mechanism and path of the impact of the level of digital transformation on enterprises performance are only studied from the perspective of resource-based theory and trade theory, and the agency and approach are not explored from other perspectives. Third, the endogeneity problem and the measurement of variables are not perfect.

### Opportunities for further research

Therefore, future research can extend the research on digital transformation on the performance of large and small, medium, and micro-manufacturing export enterprises, respectively; the latter study will extended from other perspectives and address the endogeneity problem and variable measurement design.
